# Treatment of an aggressive STLV-1 associated lymphoma in a naturally infected baboon

**DOI:** 10.1186/1742-4690-12-S1-P5

**Published:** 2015-08-28

**Authors:** Jocelyn Turpin, Sandrine Alais, Ambroise Marçais, Julie Bruneau, Anat Melamed, Nicolas Gadot, Olivier Hermine, Charles Bangham, Romain Lacoste, Renaud Mahieux

**Affiliations:** 1 Equipe Oncogenèse Rétrovirale, INSERM U1111 – CNRS UMR5308, France; 2 Equipe labellisée “Ligue Nationale Contre le Cancer”, INSERM U1111 – CNRS UMR5308, France; 3 International Center for Research in Infectiology, INSERM U1111 – CNRS UMR5308, France; 4 Ecole Normale Supérieure de Lyon, Lyon, 69364 Cedex 07, France; 5 Université Lyon 1, Lyon, 69364 Cedex 07, France; 6 Section of Virology, Department of Medicine, Imperial College London, London W2 1PG, UK; 7 Service d’hématologie adulte, centre de référence des mastocytoses, CNRS UMR 8147, institut Imagine, hôpital Necker Enfant Malade, 75015 Paris, France; 8 Service d’Anatomie et Cytologie Pathologiques, Faculté de Médecine et AP-HP, Hôpital Necker-Enfants Malades, Université Paris Descartes, Paris Sorbonne Cité, Paris Cedex 15, France; 9 Anipath, UFR Médecine Lyon-RTH Laennec, Lyon, France; 10 Station de Primatologie-UPS846-CNRS, Rousset sur Arc, 13790, France

## 

Human T Lymphotropic Virus type 1 infection is associated with a malignant lymphoproliferation named Adult T cell Leukemia/Lymphoma (ATLL). STLV-1, the simian counterpart of HTLV-1 also causes an ATLL-like disease. During the surveillance of our STLV-1 naturally infected Papio anubis cohort (n=45), we identified a 9 years-old female baboon exhibiting dyspnea, marked emaciation, and a lymphocyte count over 1010/L, pulmonary metastases and skin lesions similar to that observed in human ATLL patients. These symptoms suggested a hematologic malignancy induced by STLV-1. This diagnosis was confirmed by the evidence of massive lymphoproliferation in an inguinal lymph node biopsy and the presence of lymphocytes with characteristic abnormal nuclei (i.e. flower cells) in blood smears. As is the case for humans, the animal received a combination of AZT (Combivir) and alpha interferon (viraferon, 50 μg/week) for 4 months. During this period, blood proviral load (PVL) was measured every week. Due to the absence of health improvement and only a slight decrease in the PVL, the animal was euthanased. Histological analysis of the secondary lymphoid organs was performed, and PVL was measured in 25 different organs. All lymphoid organs contained CD3+, CD25+ lymphocytic infiltrates. Furthermore, lymphoma cells were found in lungs and liver. Tax-positive cells were detected by immunohistochemistry in spleen, lung, mesenteric, axillary, inguinal, tracheo-bronchial, lymph nodes. While all organs were PCR positive, the highest PVL was found in lymph nodes, spleen and lungs. Finally, the oligoclonality and the clonal diversity was analyzed in PBMCs throughout the treatment but also in the different organs. In conclusion, non-human primates naturally infected with STLV-1 represent a useful model to study viral pathogenesis and to evaluate new treatments.

